# Embodied navigation: the influence of lived experience on physical activity and sedentary behavior among older adults

**DOI:** 10.1080/17482631.2024.2313657

**Published:** 2024-02-13

**Authors:** Joakim Niklasson, Sofia Backåberg, Terese Lindberg, Patrick Bergman, Cecilia Fagerström

**Affiliations:** a Faculty of Health and Life Sciences, Linnaeus University, Kalmar, Sweden; b Faculty of Health, and Life Sciences, Växjö, Sweden; c University of Calgary, Faculty of Kinesiology, Linnaeus University, Calgary, Canada; d Blekinge Institute of Technology, Department of Health, Karlskrona, Sweden; e Faculty of Health and Life Sciences, Department of Medicine and Optometry, eHealth Institute, Linnaeus University, Kalmar, Sweden; f Faculty of Health and Life Sciences Kalmar, Sweden and Department of Research, Region Kalmar County, Linnaeus University, Kalmar, Sweden

**Keywords:** Acceptance, body awareness, healthy ageing, life course perspective, lived experience, physical activity, sedentary behaviour

## Abstract

**Purpose:**

The impact of a sedentary lifestyle on health and well-being is well recognized. However, there is limited understanding of how a lifetime of physical activity and sedentary behaviour influences an active lifestyle in older adults. The aim of this study was to describe how lived experience of physical activity and sedentary behaviour impacts daily activities among older adults, from a life course perspective.

**Methods:**

Qualitative content analysis was used; individual telephone interviews were conducted with fourteen older adults aged 71 to 92 years. The participants received initial support from community care and lived in ordinary housing in southern Sweden.

**Results:**

The interviews yielded one theme, “Navigating with an embodied activity compass,” and two sub-themes: “Being guided by the past” and “Unveiling pathways through body awareness.”

**Conclusions:**

Our study highlights how older adults’ lived experiences of physical activity, with their connections to body awareness and acceptance, impact daily physical activity. These findings offer new knowledge for clinical practitioners balancing recommendations of sedentary behaviour and physical activity, to promote healthy daily physical activity among older adults. Future research and policies should consider the lived experiences of older adults when addressing public health matters related to sedentary behaviour and physical activity.

## Introduction

Remaining physically active in old age is a challenge that has attracted substantial research interest, and sedentary behaviour has been included in that discussion in recent years (Katzmarzyk et al., 2019; Lachman et al., 2018; Pate et al., 2008; Rezende et al., 2014; WHO, 2020). Today, there are knowledge gaps regarding when sedentary behaviour becomes detrimental for health leading to uncertainty in recommendations on physical activity (Stamatakis et al., 2019). Older adults do not spend more time sedentary than other age groups, but with reduced physical resources and increasing impairments, the impact of sedentary behaviour is more likely to result in a physically inactive lifestyle (Bull et al., 2020; Chastin, Fitzpatrick, et al., 2014; Chastin, Mandrichenko, et al., 2014; Harvey et al., 2015; Lachman et al., 2018). Thus, understanding sedentary behaviour and encouraging physical activity becomes an area of interest when promoting healthy ageing among older adults (WHO, 2015, 2020).

Older adults are recommended to engage in at least 75–150 minutes of vigorous physical activity or at least 150–300 minutes at moderate physical activity per week and to perform resistance training twice weekly to improve their balance (WHO, 2020). Regarding sedentary behaviour, the focus in the recommendations is that older adults should spend as little time as possible sedentary, by replacing sedentary activities with physical activity (WHO, 2020). Increasing the amount of physical activity in daily life is not always an easy task, and a great challenge lies in overcoming existing barriers to physical activity (Chastin, Mandrichenko, et al., 2014; Chen, 2010; Gray et al., 2016; Spiteri et al., 2019). Sedentary behaviour and physical activity among older adults are complex and interwoven with both meaning and perceptions of sedentary behaviour, which have been shown to overlap with being physically active (Compernolle et al., 2019; Niklasson et al., 2023). In the effort to understand physical activity behaviour, there is a need to understand why older adults perform sedentary behaviour, since the variety in behaviour outcome indicates that choice of behaviour is more than just a result of becoming older (Compernolle et al., 2019; Leung et al., 2017; McGowan et al., 2021; Niklasson et al., 2023; Palmer et al., 2021). This leaves knowledge gaps regarding how the experiences acquired throughout the lifespan impacts attitudes to physical activity and sedentary behaviour later in life (Compernolle et al., 2019; McEwan et al., 2016; McGowan et al., 2021; Niklasson et al., 2023). With the overarching goal to promote healthy ageing, the aim of this study was to describe how lived experiences of physical activity and sedentary behaviour acquired throughout a lifespan, affect daily physical activity among older adults.

## Materials and methods

### Design

This study had a qualitative design based on individual semi-structured interviews, analysed in accordance with Graneheim et al. (2017); Graneheim and Lundman (2004); Lindgren et al. (2020), focusing on gaining new understanding of physical activity-related choices based on the participants’ lived experience.

### Participants

The participants were community-dwelling individuals aged 71–92 years [mean: males 84 years, females 86 years] living in a municipality in southern Sweden (Table I). They were recruited from a larger, cross-sectional questionnaire study, *Sedentary behavior in older adults and supportive methods to promote healthy aging*. The recruitment procedure has been explained in detail in a previous study (Niklasson et al., 2023). To be included in the current study, the participants had to receive initial support, defined as security alarms and/or food distribution from municipal caregivers. Individuals with a known cognitive impairment were excluded before recruitment, as a part of the above mentioned cross-sectional questionnaire study’s exclusion protocol. All participants who filled out the baseline questionnaire were given the opportunity to participate in the current study and out of the thirty older adults who showed interest, fourteen were randomly allocated to this study.

**Table I.** Participants.

Id	Social status and residence	Sex	Indoor physical activity	Outdoor physical activity
1	Cohabiting in an apartment	Male	With the support of a cane	With support of cane
2	Living alone in an apartment	Female	Without walking aids	With support of walker
3	Living alone in an apartment	Female	Without walking aids	No assistive device
4	Cohabiting in a house	Male	With support of a walker	With support of walker
5	Living alone in a house	Male	Without walking aids	No assistive device
6	Living alone in a house	Male	Without walking aids	No assistive device
7	Living alone in a house	Male	With support of a walker	With support of walker
8	Living alone in an apartment	Male	With support of a walker	With support of walker
9	Living alone in an apartment	Female	With support of a walking table	With support of another person
10	Cohabiting in a house	Female	Without walking aids	No assistive device
11	Living alone in an apartment	Male	Without walking aids	With support of walker
12	Living alone in an apartment	Female	Without walking aids	With support of walker
13	Living alone in a house	Male	Without walking aids	No assistive device
14	Living alone in a house	Female	Without walking aids	No assistive device

### Interviews

The interviews were conducted between January 2021 and May 2021 by interviewers JN and TL. JN has clinical expertise within occupational therapy, TL within caring science, and both interviewers have expertise regarding in-depth interviews. The interviews were recorded and transcribed verbatim.

Each interview opened with the question “What comes to mind when I say sedentary behavior?” followed by probing questions focused on the participant’s lived experiences, which created a reflective habitat for the interviews (Kvale, 1996). The interviewees’ stories were continuously confirmed through summarizing, and conscious pauses were made by the interviewers during the interviews to ensure that the interviewees were uninterrupted. To confirm the interviewees’ stories, mirroring brief summaries were continuously discussed throughout the interviews. Aligned with the guidance of Lindgren et al. (2020), the total number of participants was determined when no new information appeared. The length of the interviews varied, with the longest being 54 minutes and the shortest 17 minutes (average 33 minutes). Both interviewers strived to capture the lived experiences. Due to the aftermath of restrictions related to the COVID-19 pandemic, all interviews were held over the phone.

### Data analysis

To obtain rigour the *Consolidated criteria for reporting qualitative research (CCOREQ) (Tong et al., 2007)* was used. NVivo (LTD, 2020) was used to structure the interview text. In the first step of analysis, the entire text was read repeatedly to get a sense of the whole, and JN, SB, and TL shared a preliminary analysis within the group, with interpretations being discussed. Then, meaning-bearing units connected to the aim were identified and condensed. The condensed meaning-bearing units were coded and grouped into categories. The categories were discussed with all authors to ensure that interpretation was avoided and that the categories remained close to the original text. Lastly, the theme and subthemes were formulated with the ambition of visualizing the underlying thread of meaning originating from the meaning-bearing units..

Dialogues regarding interpretation were conducted over time, striving for consensus. Finding consensus was an ongoing process of ensuring that the interpretations of the text were relatable and contextualized. Thus, this was a process of moving back and forth between understanding the parts of the text and rebuilding the parts into whole passages of text. To ensure that preunderstandings of physical activity and sedentary behaviour among older adults did not dominate the analysis, continuous dialogue was conducted within the research group.

## Results

The analysis yielded one main theme, “Navigating with an embodied activity compass,” with two sub-themes, “Being guided by the past” and “Unveiling pathways through body awareness,” and four categories: “Replicating old habits,” “Wanting to be as physically active as before,” “Having insight into limitations fosters acceptance,” and “Making conscious choices by understanding the body’s capabilities” (Table II). Navigating meant making conscious choices based on what one believed was best for one’s own health and well-being. These conscious choices, built on the guidance of lived experience of physical activity and sedentary behaviour, gave the older adults the final say regarding entitlement to support in daily physical activity and the actual need of such support. An older adult’s daily route on the map of life was guided by an embodied activity compass, formed by two parts: finding guidance in experiences gained from a lifetime of physical activities, and choosing paths in life by listening to the lived body’s narrative.

**Table II.** Overview of theme, subtheme, and categories.

Theme	Sub-theme	Category
Navigating with an embodied activity compass	Being guided by the past	Replicating old habits Wanting to be as physically active as before
	Unveiling pathways through body awareness	Having insight into limitations fosters acceptance Making conscious choices by understanding the body’s capabilities

### Being guided by the past

The older adults took guidance from the past based on satisfaction or dissatisfaction with how and why physical activities had been undertaken throughout life. Satisfaction came from the body performing physical activities as it always had, whereas dissatisfaction arose when the older adults felt less active than before. The older adults felt a strong urge for physical activity and recognized the importance of experiencing both an active and sedentary lifestyle when making choices in their current life circumstances. Remembering past experiences gave guidance, expressed as a determination to maintain a physically active life, function, and independence.

#### Replicating old habits

For the participants, being active was ingrained as a behaviour from early life, shaping autonomous routines that influenced choices in daily physical activities as they grew older. Experiences of being sedentary or physically active in youth guided choices and reasoning later in life. Being sedentary reflected a life governed by interests and physical limitations, shaping decisions regarding daily physical activity. The excitement or personal development found in sedentary activities often led to physical activity being neglected and considered less valuable over time.

Yes, I have not been highly active before … (laughs) … either. So there have not been any sports or anything like that, I am just interested in watching sports…(laughter)…I just have not been that interested in being active. (Participant 1)

If physical activity had been initiated early in life, the older adults’ daily routines were guided by a willingness to move their body. A feeling of having tasks that one needs to accomplish on a daily basis was based on previous experience of physical activity, making physical movement an inseparable part of existence. Daily decisions were reflections of prior choices and preferences in life regarding physical activity, from early life and throughout adolescence, young adult life, and older age. Among those who had lived with physical activity as a part of everyday life, daily choices and priorities was strongly influenced by this. Being physically active was considered something particularly important, and it was clear that the experience of being physically active throughout life gave insight regarding the difference between being physically active and working out.

I mean, I have had a good childhood, and we have always been active. Not working out, but we always walked in the forest, and my wife and I always take our bikes to go shopping. (Participant 2)

#### Wanting to be as physically active as before

The older adults established their own standards for daily physical activities, based on their own physical and sedentary activities. Falling short of these self-imposed standards created a sense of uncertainty, as they lost sight of their previous way of life. This uncertainty led to the experience of living an unfamiliar daily life. The older adults pushed themselves to be active, driven by a powerful desire to regain a previous level of independence. This sense of independence not only affected healthy daily physical activity, but also influenced the risks they were willing to take. Despite being aware of the daily risks they faced, the inherent drive to be physically active prevailed. The aspiration to be as physically active as before clashed with the limitations and restrictions imposed by the ageing body. These limitations were viewed as mere obstacles to be overcome, regardless of current physical capabilities. However, drawing from past experiences of physical activity, the older adults could deviate from their previous standards if these were deemed too risky. This meant ceasing to fight against their body and accepting support from family, friends, and caring aids to continue being as physically active as wanted.

But you try as much as possible to go without it [walker], and I do, periodically I go without it a lot, but then when I feel that now I cannot, I take the walker. I have passed out a couple of times here and ended up lying down. (Participant 6)

Changes in the older adults’ standards also appeared to be influenced by the intersection of their lifetime experience of physical activity and the fear of potential injuries. The experience of fear often took precedence, leading the older adults to more sedentary activity patterns. Any inability to move as they once could prompted the older adults to reflect on recent events. Despite experiencing a shift in physical function, they maintained a positive outlook. The willingness to be physically active reflected a striving for maximum independence, driven by the motivation to continue living at home autonomously. If they were able to live independently, the older adults did not experience any lack in their daily physical activity or any need for assistance. Living in their own home, within their familiar neighbourhood, and surrounded by their personal belongings, served as a benchmark for maintaining autonomy in daily physical activity.

I live in a bungalow, and I certainly don’t want to move out of here, I know it’s almost time to do that … as long as I can … I have said this before, I want to live here until they need to take care of me in a nursing home. (Participant 9)

### Unveiling pathways through body awareness

Balancing the right to receive support in daily physical activity with the actual need for this involved a process of trusting or not trusting the body’s abilities, which was influenced by past experiences of physical activity and sedentary behaviour. Drawing from a life of physical activity, the older adults could understand the body’s limitations. This allowed them to listen to the voice of acceptance and navigate life by adapting physical activity accordingly. Through ongoing evaluations of their physical capabilities, the older adults gradually developed trust in the body’s abilities. This insight into their capabilities enabled them to assess the achievability of different physical activities. They continuously updated their understanding of the body’s capabilities, considering both possibilities and limitations when planning and engaging in daily physical activity.

#### Having insight into limitations fosters acceptance

Being limited in daily physical activity required adapting to change, managing decreased energy levels, and finding satisfaction with the trajectory of life. Older adults who had had an active lifestyle were not taken aback by the effects of ageing on physical capabilities. Their lifelong engagement in physical activity provided them with insights into the body’s limitations and helped them come to terms with the feeling of being overlooked by society. The older adults’ ability to adjust to life’s challenges was shaped by their active participation in physical activity. An advantage of a lifelong commitment to physical activity was gaining an understanding of the body and embracing the inevitability of age-related changes. Although it was challenging to cope with the increased energy demands of their ageing body, they recognized it as a natural consequence of older age. Consistently engaging in physical activity throughout life gave them solace. If physical limitations prevented them from being active, they found comfort in adopting a more sedentary lifestyle. Living a physically active daily life allowed them to perceive ageing as a natural process of transformation while still maintaining a sense of vitality.

Of course, it has changed, and I feel that … the older you get, the less you can do, but I think that, for eighty-seven, I am quite energetic … (laugh) … (Participant 14)

Doing the things one was capable of and not worrying about expectations was the core of a harmonious existence. However, even when the participants felt they had achieved what was needed, they had a persistent feeling of being lazy. This did not affect them in a negative way, as it was portrayed in the light of knowledge gained regarding the body’s capacity and needs.

Ugh … now I have … I almost said that … I do not need to be as active, because I can still do what I have to. It is pure … pure laziness, I would think. (Participant 13)

#### Making conscious choices by understanding the body’s capabilities

The older adults’ lifelong connections between the body and physical activity fostered an understanding of capabilities based on the pursuit of joyful physical activity and a sense of comfort and safety while moving. Joy, comfort, and feeling safe served as ongoing motivations for being active. If any of these foundations was compromised, a previously cherished physical activity could be deprioritized swiftly. Being cautious was ingrained in the older adults’ approach to daily physical activity, reflecting a well-established relationship with being physically active. By trusting the body’s abilities, the older adults found guidance in daily pursuits. Even if they occasionally could not fulfil their daily plans, this was usually due to conscious choices rooted in a healthy connection with the body. Being active brought immense satisfaction and a sense of wholeness. Therefore, an active lifestyle was always pursued, even if the ageing body limited possibilities compared with in the past. The pursuit of happiness through physical activity was not tied to a specific physical activity or sport, as the relationship with being active was on a spiritual or existential level. If the active aspect of a previously prioritized physical activity could no longer be pursued, the older adults no longer considered it to be worth their while.

Yes, that is why…the charm of golf is that you walk and plan your next shot while you walk some more and talk to someone. But then you have to get in a golfcart…no, ugh, no, I tried that for two years and it did not work … (sigh) … I am not able to walk as much nowadays. If you play golf, you do eighteen holes and no less. I have found other ways to walk and socialize. (Participant 7)

Being comfortable was related to an experience of understanding the body’s need for rest before, during, or after physical activity. The older adults did not ignore recuperation in their daily physical activities, regardless of intensity level. Being comfortable by relying on such rest was a conscious choice, a natural reaction, creating a life where physical activity and sedentary behaviours were interwoven as dependent on one another.

## Discussion

Navigating with an embodied activity compass allowed the older adults to find new ways to behave in daily physical activity, relying on the guidance and knowledge gained from a life of engagement in physical activity and sedentary behaviours. Past experiences of physical activity were crucial for the attitude towards sedentary behaviours later in life, showing that the past guided the older adults’ thoughts and feelings regarding acceptance and adaption to changes in life. This created a habitat that embraced changes and adaption, but the older adults who had had physical activity or sedentary behaviours embedded in daily life would strive to keep on living in the same manner as they had always done.

In a study published by Zhang et al. (2022), past life experiences were highlighted as impacting participation in physical activity, aligned with our findings regarding bringing previous routines to daily physical activity as an older adult. However, our findings indicate that routines regarding sedentary behaviour also continue in older age, which is important in the context of physical literacy in the perspective of a lifespan (Cornish et al., 2020). According to a systematic review by Edwards et al. (2017) physical literacy revolves around the knowledge and understanding gained throughout life regarding how important physical activity is for a healthy lifestyle, which in our findings serve as a lens through which the older adults see their ageing body. In the context of physical literacy, this lens is known as competence, and described as “body awareness” in our findings. Our study also shows that the older adults found acceptance through body awareness. This aligns with the findings in a previous study by Niklasson et al. (2023), where the process of becoming older was shown to be a transformational stage of adopting a more sedentary lifestyle, with the outcome of finding new paths in life. Thus, a life of physical activity eased the process of adapting to a more sedentary lifestyle.

In this study, having the ability to adapt to changes was found to be based on how well-developed an older adult’s body awareness was. Where underlying factors, such as reduced energy levels and physical impairments, prevented familiar movement patterns, knowledge regarding one’s limitations and abilities was key to acceptance. In our findings, the process of acceptance was dependent on feeling safe and comfortable during physical movement, and also enjoying the chosen physical activity. It is important not to overlook the impact of acceptance—in the sense of being satisfied with one’s existence—as it is connected with experiencing life fulfilment as an older adult (Morgan et al., 2019).

Our findings show that the older adults had a need for physical movement, a need based on the aspiration to be as capable as they had always been and staying as physically functional as possible. As mentioned by Lin et al. (2020), older adults who more often engage in physical activity tend to have a higher sense of responsibility for their own health than older adults with lower physical activity engagement. Our study adds two points of interest. Striving to be as capable and independent as wanted could cloud one’s judgement, putting the older adults at risk. However, the guidance gained from a life of physical activity revealed a path leading to balance between wanting to move more than the body allowed and the need to move enough to be independent. Striving for independent living was shown to be a force to reckon with, especially with physical activity seen through the lens of successful ageing, as independent living was a common target in older adults’ motivational processes (Lin et al., 2020). Wanting to be independent requires acceptance of changes in physical capacity and a resulting, more sedentary lifestyle. Our findings of such efforts to be independent, based on having insight into how to optimize physical capacity, combined with compensating for bodily changes, mirror the three premises of successful personal development (Baltes & Baltes, 1990). Personal development is a lifelong process that involves continuous adaptation and growth, influenced by both internal factors, such as motivation and self-regulation, and external factors, such as social support and environmental opportunities (Baltes & Baltes, 1990). In light of the characteristics of successful personal development, our findings show that the older adults’ lived experiences might play a crucial role in healthy ageing.

It is important to highlight that not all older adults have the motivation, opportunity, or capability to be physically active (Meredith et al., 2023). Our findings regarding conscious choices take age-related deterioration into consideration and do not mean that older adults always had the capability, opportunity, or motivation to be physically active. Making conscious choices was related to understanding the body’s potential and consciously adapting to this potential in performing daily physical activity as one saw fit. Furthermore, our findings show that the impact of bodily changes related to ageing could change the prioritization of physical activities that a person had previously engaged in. That bodily degeneration impacts older adults’ physical activity is nothing new (Liu et al., 2021; Rivasi et al., 2020). However, our findings reveal that new paths to discover enjoyable physical activities could be found on the map of life, through the guidance of an embodied activity compass.

### Methodological considerations

The requirements for participation in the study were carefully selected to achieve a representative sample with a variation in socioeconomic status, age, sex, and physical ability, to increase the dependability of the results (Lincoln & Guba, 1985). All older adults in this study were able to move around independently, with or without assistive device. Thus, there were no severe physical disabilities restricting daily physical activity. Due to the exclusion criteria, the participants had not developed known cognitive impairment at the time of the interviews. Because the participants in this study were in the early stages of age-related deterioration, it is important to note that our findings may not apply to all older adults. However, our findings can be applied to older adults who receive initial support from community care and live in ordinary housing, which strengthens the transferability of the study (Lincoln & Guba, 1985). Credibility was established by ensuring that the interviews were done when the interviewees story was told, a strive to rise above potential preconceptions. Continuous peer debriefing were conducted within the research group to reduce the risk of assumptions during the analysis, which strengthens the credibility of the study (Lincoln & Guba, 1985). Furthermore, multiple theoretical perspectives were used in the process of triangulating the findings, adding more strength to the credibility and confirmability to the study (Lincoln & Guba, 1985). By involving multiple researchers in interpreting and validating the data, the process was in line with the recommendations of Graneheim and Lundman (2004).

To gain depth in the interviews, it was crucial that participants had lived experiences of physical activity (Husserl & Carr, 1978). As the group consisted of older adults who could still move around independently indoors, findings included experiences of living a life filled with physical activity and how these experiences affect bodily changes in life. The lived experience of becoming more inactive due to age-related decline would also be ingrained in their surroundings, as all participants received initial support from community care.

The two interviewers followed the same interview guide with open-ended questions to adhere to the same topics, but gave space to free narratives by following up the interviewees’ response with probing questions such as *How did this make you feel? Can you expand on that? Can you give an example? What do you think is causing this?* or *What do you mean by … ?* Enabling this type of interview atmosphere is in line with the recommendations for gaining depth in the interviews (Graneheim et al., 2017). All interviews were conducted within a small window of time (three months) to reduce the risk of time-related changes and when all fourteen interviews were conducted and transcribed, the two interviewers compared their overall interpretations of the texts. When the interviewers felt that the reading and interpretation revealed repetitive findings towards the end, the findings were brought to the research group. The whole group discussed different ways to interpret the text, which was compared to the collected data when forming meaning-bearing units, categories, and themes. All included quotes were read by the research group and confirmed as corresponding to the data. The interpretation process was aimed at providing systematic understanding and analysis of data.

## Conclusions

This study underlines the importance of older adults’ lived experiences of physical activity and sedentary behaviour, especially in regard to daily physical activity. The older adults’ relationships with past physical activity and body awareness strongly influenced engagement daily physical activity. Older adults with a history of physical activity strived to maintain an active lifestyle, but sedentary behaviour could still persist in older age. Striving for independence acted as motivation in maintaining physical activity, but could sometimes lead to risk-taking. However, a lifetime of engagement in physical activity gave guidance in balancing desired level of physical activity with physical capabilities. The older adults’ abilities to adapt to changes were closely linked to their body awareness, and understanding limitations and capabilities was crucial for making conscious choices. Engagement in physical activity throughout life brought knowledge that helped the older adults navigate through the changes and challenges related to an ageing body.

These findings could be used by clinical practitioners as guidance regarding daily physical activity for older adults living in ordinary housing, receiving initial support from community care, especially regarding balancing recommendations of sedentary behaviour and physical activity. Future research targeting vital public health matters related to a healthy physically active lifestyle of older adults, should include lived experiences. For policies regarding healthy ageing, the lived experience should be included when weighing the impact of increasing physical activity levels in the daily life of the older adult.

## Data Availability

The authors confirm that the data supporting the findings of this study are available within the article [and/or] its supplementary materials.

## Appendix

**Interview Guide:**

1. What are your thoughts on sedentary behavior among older adults?
2. Can you describe how you move around during a typical day?
3. Can you describe how comfortable you feel with moving around in your daily life?
4. How confident do you feel in moving around in your daily life?
5. What do you think could help you feel more secure or comfortable in your movements in the future?
6. What would you do differently in your daily life if you felt more secure or comfortable in your movements?
7. How do you perceive that your level of physical activity has changed throughout your life? How do you think your past experiences with physical activity influence your sedentary behavior today?

**Examples of narrative probing questions:**

How did this make you feel?
Can you elaborate?
Can you provide an example?
What do you think causes this?
What do you mean by...?
What does it mean to you when you say...?

**Arching research questions to have in mind during the interview:**

What do older adults perceive to affect their ability to move around in their daily lives? How do older adults perceive that their past experiences with physical activity throughout their lives influence their sedentary behavior today?
